# Exodermis and Endodermis Respond to Nutrient Deficiency in Nutrient-Specific and Localized Manner

**DOI:** 10.3390/plants9020201

**Published:** 2020-02-06

**Authors:** Jiří Namyslov, Zuzana Bauriedlová, Jana Janoušková, Aleš Soukup, Edita Tylová

**Affiliations:** Department of Experimental Plant Biology, Faculty of Science, Charles University, Vinicna 5, 128 44 Prague 2, Czech Republic

**Keywords:** exodermis, nutrient deficiency, nitrogen, Casparian bands, suberin lamellae, split-root cultivation, maize, barley, high-affinity transporters

## Abstract

The exodermis is a common apoplastic barrier of the outer root cortex, with high environmentally-driven plasticity and a protective function. This study focused on the trade-off between the protective advantages provided by the exodermis and its disadvantageous reduction of cortical membrane surface area accessible by apoplastic route, thus limiting nutrient acquisition from the rhizosphere. We analysed the effect of nutrient deficiency (N, P, K, Mg, Ca, K, Fe) on exodermal and endodermal differentiation in maize. To differentiate systemic and localized effects, nutrient deficiencies were applied in three different approaches: to the root system as a whole, locally to discrete parts, or on one side of a single root. Our study showed that the establishment of the exodermis was enhanced in low–N and low–P plants, but delayed in low-K plants. The split-root cultivation proved that the effect is non-systemic, but locally coordinated for individual roots. Within a single root, localized deficiencies didn’t result in an evenly differentiated exodermis, in contrast to other stress factors. The maturation of the endodermis responded in a similar way. In conclusion, N, P, and K deficiencies strongly modulated exodermal differentiation. The response was nutrient specific and integrated local signals of current nutrient availability from the rhizosphere.

## 1. Introduction

The exodermis is the apoplastic barrier of the outer root cortex [1], common in seed plants [2,3]. Its differentiation is enhanced under various stress factors, e.g., drought, salinity, toxicity of heavy metals, organic acids, or sulphides [4,5,6,7,8,9,10], which is similar to the response of endodermis [10,11]. Due to its position in root cortex, the exodermis is very important for the protection of internal tissues. The presence of the exodermis prevents the uptake of harmful compounds from the rhizosphere [7,12,13,14] and also modulates radial water and nutrient transport [7,15,16]. Although similar to the endodermis, the exodermis exerts higher evolutionary variability and responsiveness to environmental inputs [1,17,18,19].

Although it is expected that differentiation of the exodermis would influence nutrient uptake, there is currently limited information on how nutrient availability governs developmental plasticity. Exodermal Casparian bands (CB) and suberin lamellae (SL) limit apoplastic transport deeper into the cortex [4] and significantly reduce the surface of cortical plasmalemma available for nutrient uptake to the symplast [20,21]. This implies that a less differentiated exodermis might be more favorable under nutrient deficiency. The position of the exodermis might also modify the role of the endodermis in regulation of nutrient uptake.

Endodermal differentiation/suberization was reported to be enhanced under –K or –S stress, but delayed under –Fe, –Mn, or –Zn stress in roots of *Arabidopsis thaliana* grown on agar plates [22,23]. Root absorption characteristics change during the transition of the endodermis from first stage of differentiation (with developed CB) to second stage (with deposited SL). During this transition, the endodermis functionally switches from an absorptive role to a protective role. [24,25,26]. The distribution of root membrane transporters fine tunes nutrient acquisition and balances the apoplastic and symplastic transport pathways [25,27]. Nutrient uptake and radial transport are thus precisely optimised across changing nutrient availability by combining fast delivery of nutrients to vascular tissues under non-limiting conditions with the effective nutrient acquisition under limiting conditions [27]. However, what impact does the exodermis have? Does exodermal differentiation into a protective later counteract the effectivity of nutrient uptake? While its peripheral position might predict this, published studies show inconsistent and non-systemic results. These results were mostly obtained in plants grown in hydroponic cultures. Roots of *Ricinus communis* delayed exodermal suberization under NO_3_^−^ deficiency [20]. Rice roots responded to high NH_4_^+^ with enhanced lignification and suberization of apoplastic barriers [28]. Three wetland *Carex* species (*C. vesicaria*, *C. rostrata*, and *C. gracilis*) showed enhanced exodermis differentiation in oligotrophic compared to eutrophic conditions in sand culture [29]; enhanced exodermal suberization was found in maize under Mg deficiency [30]. Armand (2019) recently emphasised that enhanced endodermal and exodermal differentiation in low-N and low-P barley corresponds with reduced root hydraulic conductivity [31].

In this study, we analysed the effect of nutrient deficiency on exodermis differentiation in maize roots, which have been commonly used in studies focusing on root structure and function. We exposed maize seedlings to selected nutrient deficiencies (N, P, K, Ca, Mg, Fe) to test the exodermal response to nutrient limiting conditions, and whether they affect the whole root system or only localized sections. Besides maize, we also included barley in the study and analysed its response to P deficiency and detected the localization of high-affinity Pi transporters in its roots. Our results show that the response to deficiency is nutrient-specific and that plants integrate the actual nutrient availability of the rhizosphere on the scale of individual roots to fine-tune the exodermal differentiation process.

## 2. Results

### 2.1. Exodermis Differentiation under Nutrient Deficiency in Maize Roots

**Figure 1:**
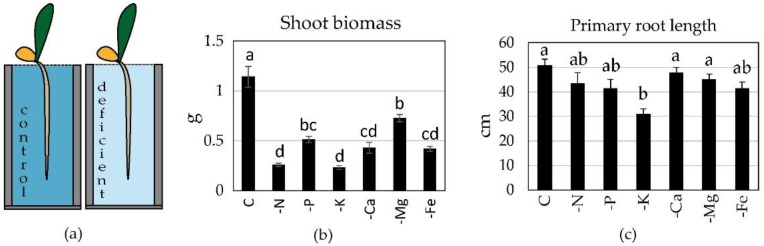
Maize growth in deficiency treatments. (**a**) The arrangement of the culture - deficiency applied to whole root system; (**b**) Shoot biomass (dry weight) and (**c**) primary root length of plants (mean ± SE, n = 5–8) after 14 days of cultivation. Treatments: control (C), deficient (-N, -P, -K, -Ca, -Mg, -Fe), each completely lacking the particular nutrient. Different letters indicate significant differences among treatments (One-way ANOVA, Bonferroni test, *p* < 0.05).

Deficiency of selected nutrients (N, P, K, Ca, Mg, or Fe) were representative of common rhizosphere conditions. When applied to the whole root system, maize seedlings cultivated for 14 days in hydroponic conditions (Figure 1a) showed visible symptoms of deficiency (e.g., leaf chlorosis, deformations, drying, or purple coloration). This indicated that the deficiency treatments had an effect on overall plant growth, which manifested itself as lower shoot biomass compared to the controls (Figure 1b). Only minor changes in the length of primary roots were recorded among treatments, the only statistically significant result was for K deficiency (Figure 1c).

**Figure 2:**
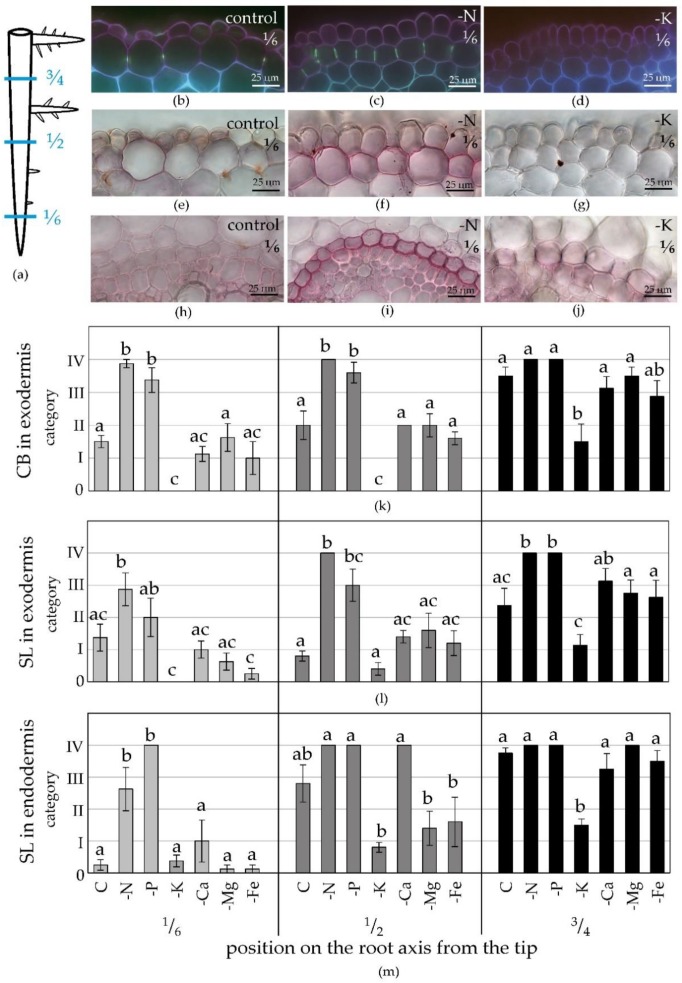
The effect of nutrient deficiency on differentiation of maize exodermis and endodermis. (**a**) The positions on the root axis subjected to anatomical analyses; (**b**–**d**) Exodermal Casparian bands in (**b**) control, (**c**) –N, and (**d**) –K roots; (**e**–**g**) Exodermal suberin lamellae in (**e**) control, (**f**) –N, and (**g**) –K roots; (**h**–**j**) Endodermal suberin lamellae in (**h**) control, (**i**) –N, and (**j**) –K roots. Berberine-Crystal violet staining, UV (**b**–**d**), Sudan Red 7B staining (**e**–**j**); (**k**–**m**) The establishment of (**k**) exodermal Casparian bands, (**l**) exodermal suberin lamellae, and (**m**) endodermal suberin lamellae at positions ^1^/_6_, ½, or ¾ of the root axis from the tip (mean ± SE, n = 5–8). Treatments: control (C), deficient (–N, –P, –K, –Ca, –Mg, –Fe), each completely lacking the given nutrient. The category 0-IV indicates the incidence (%) of cells with CB or SL within the layer. Categories: 0 (0%); I (<30%); II (±50%); III (>70%); IV (100%). Different letters show significant differences among treatments (One-way ANOVA, Bonferroni test, *p* < 0.05).

We analysed root anatomy at several positions along the axis of the main root (Figure 2a). The nutrient deficiencies significantly affected the differentiation of root apoplastic barriers. Deficiency of N or P strongly accelerated the differentiation of the exodermis and endodermis compared to the control and other treatments (Figure 2b–m). The exodermis completed its primary and secondary developmental state very close to the root tip, and CB and SL were present in the majority of exodermal cells already at the position ^1^/_6_ of the root axis from the tip, where only few cells with differentiated CB occurred in the control treatment (Figure 2b–d,k). 

The opposite effect was found in –K roots, where differentiation of the exodermis was pronouncedly delayed (Figure 2b,d,k) and CB were not detectable before half (approx. 15 cm) of the root length (Figure 2k). Similar trends were observed for exodermal suberin lamellae deposition. The fastest transition to suberized exodermis occurred in –N and –P treatments; suberization in the –K treatment was markedly delayed (Figure 2e–g,l). Suberization of the endodermis was affected similarly to the exodermis (Figure 2h–j,m). Effects of other deficiencies (–Ca, –Mg, and –Fe) were generally mild. Fe deficiency slightly delayed the differentiation of the barriers, but the effect was not statistically significant. 

### 2.2. Localized Response to Nutrient Deficiency at the Level of the Whole Root System (Split-Root Cultivation of Maize)

**Figure 3:**
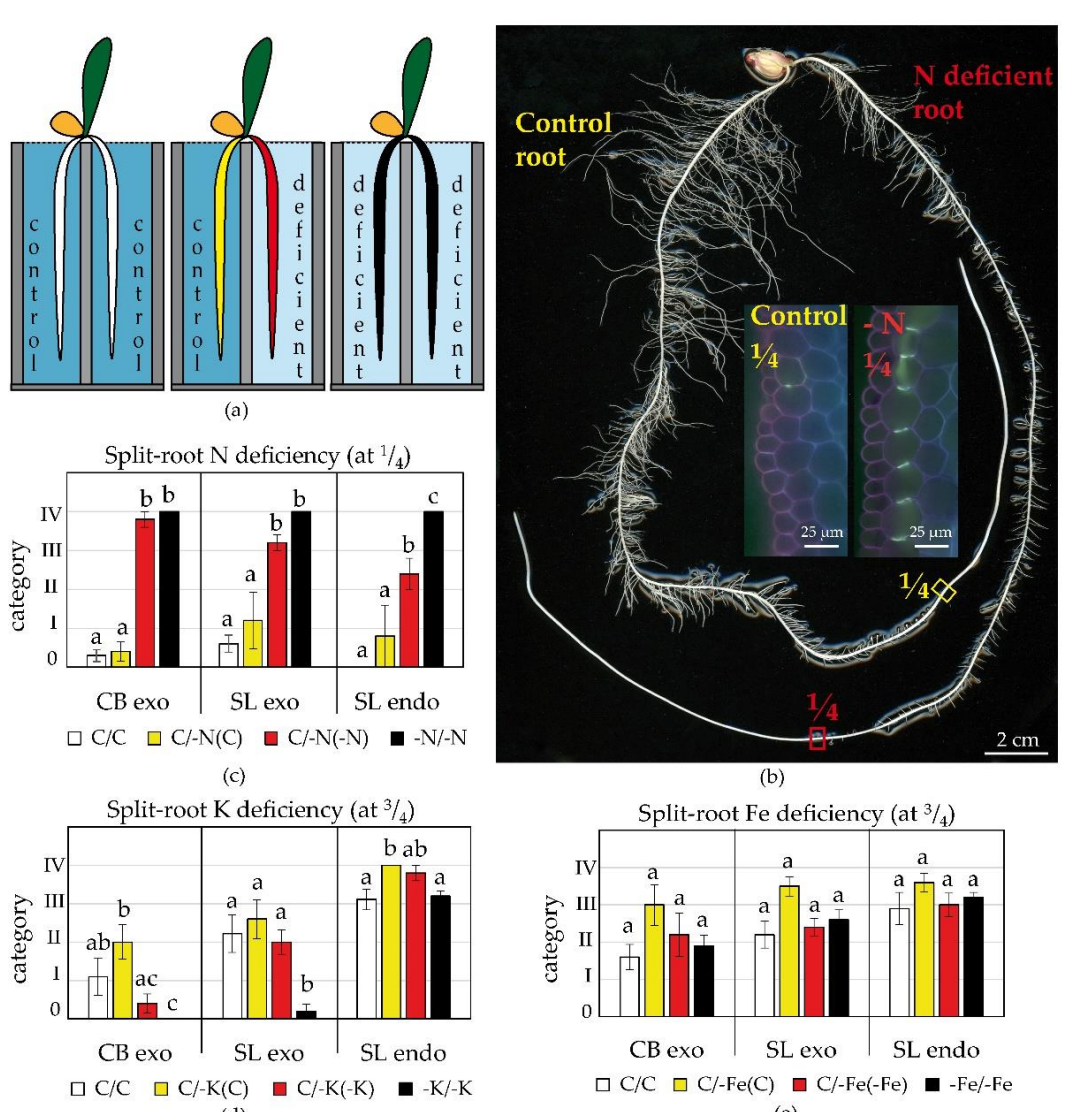
The localized effect of nutrient deficiency on differentiation of maize exodermis and endodermis in split-root hydroponics. (**a**) The arrangement of split-root cultures (colors indicating the treatments are kept in the graphs). (**b**) Appearance of roots in split-root N deficiency; insets show exodermal CB in control (left) and N deficient (right) root at the position ¼ of the root axis from the tip, Berberine-Crystal violet, UV; (**c**–**e**) The establishment of exodermal Casparian bands (CB exo), exodermal (SL exo) and endodermal (SL endo) suberin lamellae in (**c**) –N, (**d**) –K, and (**e**) –Fe roots at position ¼ (N deficiency) or ¾ (Fe, K deficiency) of the root axis from the tip (mean ± SE, n = 5). Deficiencies were applied in the following combination of nutrient solutions in split-root chambers: control/control (C/C), control/deficient (C/–N, C/–K, C/–Fe), and deficient/deficient (–N/–N, –K/–K, –Fe/–Fe). C/–N(–N), C/–Fe(–Fe), and C/–K(–K) are roots growing in the solution without the given nutrient; C/–N(C), C/–Fe(C), and C/–K(C) are roots of the same plant growing in the control solution. The category 0-IV indicates the incidence (%) of cells with CB or SL within the layer. Categories: 0 (0%); I (<30%); II (±50%); III (>70%); IV (100%). Different letters indicate significant differences among treatments (One-way ANOVA, Bonferroni test, *p* < 0.05).

We used split-root cultivation (Figure 3a) to test the local and systemic response of roots when a nutrient is available, but not acquired from the rhizosphere of a given root. Three contrasting deficiency treatments were selected according to the results of previous experiments: nitrogen deficiency, which stimulated exodermal differentiation, and potassium and iron deficiencies, which both delayed differentiation but differ in their phloem mobility and thus capacity to be redistributed.

In the split root experiment, plants with half their roots experiencing nutrient deficiency and the other half in control solution (C/–N, C/–K, or C/–Fe) produced comparable (ANOVA, *p* > 0.05) shoot biomass as the full control (C/C), but fully N or K deficient plants (–N/–N, –K/–K) were significantly smaller (ANOVA, *p* < 0.05, data not shown). This indicated that half a root system was sufficient to provide nutrient for normal shoot growth during the 10-day cultivation period. Despite normal biomass, split root plants from the C/–Fe treatment displayed a clear pattern of partial leaf chlorosis. Only half of the leaf lamina was chlorotic, indicating limited translocation of Fe within the leaf. 

In the split-root C/–N treatment, root branching was significantly enhanced in the control chamber (Figure 3b). The total length of lateral roots was 86% higher in the control chamber of C/–N treatment (384.3 and 225.4 cm in C and –N chambers, respectively; ANOVA, *p* < 0.05). A similar trend was observed in the C/-K treatment. The lateral root length was 40% higher in the control chamber of C/–K treatment (756.7 and 556.6 cm in C and –K chambers, respectively; ANOVA, *p* < 0.05). The mildest effect was observed in C/–Fe treatment. Although lateral roots were 20% longer in the control chamber, the effect was not significant (302.7 and 254.7 cm in C and –Fe chambers, respectively; ANOVA, *p* > 0.05). 

Interestingly, N deficiency applied to half of the root system induced strongly asymmetric differentiation of the endodermis and exodermis. The differentiation of the exodermis was clearly enhanced in roots growing in N deficient solution (C/–N(–N)) compared to roots in the control solution (C/–N(C)) of the same plant. The C/–N(–N) roots resembled roots of plants subjected to N deficiency in both halves of split-root cultivation (–N/–N) and had an almost fully established exodermis close to the root tip (positioned at ¼ of the root axis from the tip. C/–N(C) roots were similar to the roots of control plants (C/C) and had only few cells with CB or SL in the exodermis or endodermis at the same position (Figure 3b-insets,c). In split-root K deficiency, –K roots (C/–K(–K)) had less pronounced exodermal lignification compared to the control roots (C/–K(–C)) of the same plant (Figure 3d). In split-root Fe deficiency, –Fe root (C/–Fe(–Fe)) and control root (C/–Fe(C)) did not significantly differ (Figure 3e). Results derived from these split root experiments correspond nicely to differentiation patterns induced by homogenous deficiency treatments but highlight the localized response.

### 2.3. Localized Response to Nutrient Deficiency at the Level of a Single Root (“Sandwich” Cultivation of Maize)

**Figure 4:**
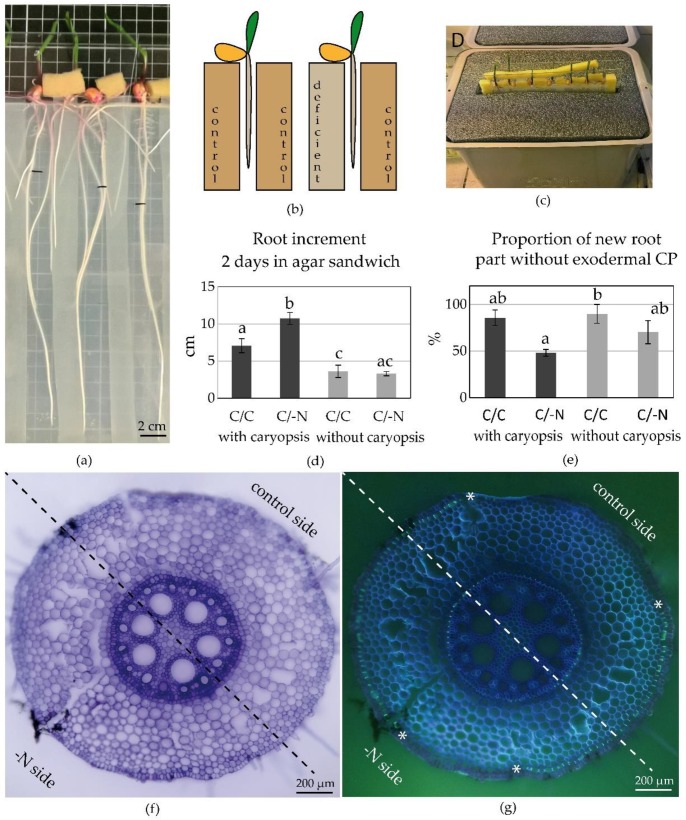
The localized effect of nutrient deficiency on differentiation of maize exodermis and endodermis in agar “sandwich” cultivation. (**a**) 6 DAG plants after 2-day cultivation between agar slabs (black marks indicate root lengths at the start of “sandwich” cultivation); (**b**) The arrangement of the cultivation; (**c**) Agar sandwich in the cultivation chamber; (**d**) Root increment within 2-day cultivation in agar. Combinations of agar slabs: control/control (C/C), control/–N (C/–N); (**e**) The proportion (%) of root part newly grown in agar and lacking established exodermal CB. Different letters indicate significant differences among treatments (mean ± SE, n = 3–5; One-way ANOVA, Bonferroni test, *p* < 0.05). (**f**,**g**) Establishment of exodermal Casparian bands in C/-N agar sandwich (root was marked by shallow cut and black ink at the time of harvest; asterisks enclose the area with exodermal Casparian bands). Berberine-Crystal violet staining, (**f**) bright-field, (**g**) UV.

The asymmetrical differentiation of apoplastic barriers observed in the –N split-root cultivation encouraged us to look more closely at the local response to nutrient deficiency in roots. To do this, we created an agar “sandwich” by placing a N deficient agar slab on one side of the primary root of 4-DAG maize seedlings and a control agar slab on the other side (Figure 4a–c). In this setup, the primary roots of plants in such control/deficient (C/–N) sandwich elongated faster (Figure 4d) and had enhanced exodermal differentiation compared to plants in control/control (C/C) sandwich (Figure 4e). A one-sided local response to N availability was however not found. We observed asymmetric differentiation of exodermis, but this response was probably caused by small air gaps between the root and the agar slabs. Lignified exodermal CB were only found below surfaces facing these air gaps, rather than either the control or -N agar slabs (Figure 4f,g). 

We further tested whether the local response to N availability would be enhanced by removal of the caryopsis (a source of N for young seedlings) over short-term cultivation. We used the same 4-DAG seedlings, but excised the caryopses just before placing the roots between the agar slabs. The caryopsis absence retarded root growth in both C/–N and C/C plants and slightly delayed exodermis differentiation in C/–N roots (Figure 4d,e), but a localized response to N availability was again not found.

### 2.4. Exodermal Differentiation Versus the Localization of Nutrient Transporters in Barley Roots 

Exodermal differentiation in the root periphery should restrict apoplastic communication between the middle cortex and the rhizosphere. Such a limitation might compromise the effectivity of nutrient transporters. To analyse the impact of exodermal differentiation on the presence of nutrient transporters in root, we introduced barley as an additional model organism in our research. This allowed us to take advantage of commercially available antibodies for high-affinity Pi transporters, which are unavailable for maize. High-affinity transporters are generally most active under deficiency conditions. 

**Figure 5:**
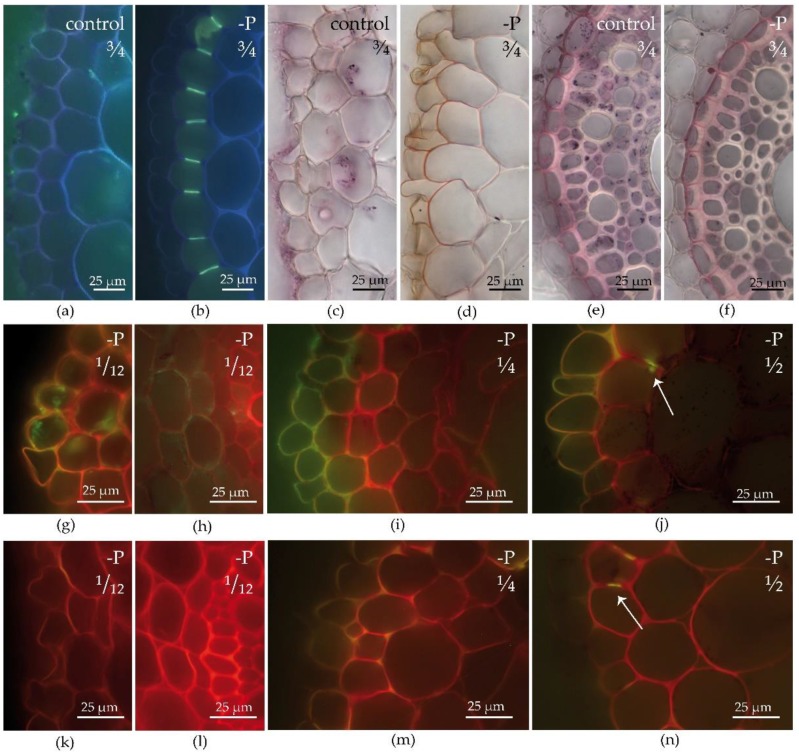
The localization of high-affinity phosphate transporters and apoplastic barriers differentiation in barley roots; (**a**–**f**) The establishment of exodermis and endodermis in (**a**,**c**,**e**) control and (**b**,**d**,**f**) –P barley roots at position ¾ of the root axis from the tip, (**a**,**b**) exodermal Casparian bands, Berberine-Crystal violet staining, UV, (**c**,**d**) exodermal and (**e**,**f**) endodermal suberin lamellae and U-thickenings, Sudan Red 7B staining; (**g**–**j**) The immunodetection of Pht1;1-2 high-affinity transporters in –P barley roots at positions (**g**,**h**) ^1^/_12_, (**i**) ¼, and (**j**) ½ of the root axis from the tip (Anti-HvPht1;1-2 primary antibody, IgG DyLight^TM^ 488 secondary antibody, Crystal violet counterstaining for quenching the autofluorescence, WB; green color indicates the signal of antibody in the pictures), (**g**,**i**,**j**) rhizodermis and part of cortex, (**h**) detail of middle cortex; (**k**–**n**) Negative controls made at same positions as (**g**–**j**), with omitted primary antibody from the immunodetection procedure. The arrows indicate lignified Casparian bands in exodermal cells.

Barley was cultivated in control and P deficient conditions. Differentiation of the exodermis was significantly accelerated in –P barley roots, similar to maize. –P plants had a partially established exodermis (a few lignified CB in the exodermal layer) at ½ of the root length and almost complete primary exodermis at the ¾ of the root from the tip (Figure 5b). In contrast, control plants completely lacked exodermal CB (Figure 5a). Similarly, suberization of the exodermis (Figure 5c,d) and endodermis was enhanced in –P roots (Figure 5e,f). 

The high affinity transporters (HvPht1;1-2) showed a clear response to P availability and distance to the root tip. Control plants did not show any signal of HvPht1;1-2 antibody at any analysed position along root axis (not shown). In contrast, positive immunostaining indicated the presence of transporters in –P roots (Figure 5g–j; negative controls without primary antibody for same sections in Figure 5k–n). Moreover, the presence of HvPht1;1-2 at cortical and rhizodermal membranes changed along root axis. At the ^1^/_12_ position along the root axis (young root, close to the tip), the transporters were present in the rhizodermis as well as the cortex, including its deeper layers (Figure 5g,h; negative controls without primary antibody in Figure 5k,l). In older root parts, the signal almost disappeared from the middle cortex. We found a strong positive antibody signal in the rhizodermis and the exodermis at the ¼ position along the root axis, but a very weak signal in the middle cortex (Figure 5i; negative control without primary antibody in Figure 5m). In some sections, we even observed an asymmetric distribution of transporters within the exodermal plasmalema (in the outer tangential plasmalema domain only, not in the inner one; Figure 5i). In older root sections, the signal was detectable only in the rhizodermis or was completely missing (Figure 5j; negative control without primary antibody in Figure 5n). We detected the first lignified CB at the ½ position along the root axis (Figure 5n). The observed redistribution of transporters thus preceded the lignification (detectable with the use of histochemistry) of exodermal CB to some extent. 

## 3. Discussion

### 3.1. Nutrient Deficiency Affects Differentiation of Apoplastic Barriers in a Nutrient-Specific Manner

Plants are sessile organisms and their fitness is related to their high developmental plasticity and their ability to adjust resource acquisition to local conditions. Root system architecture, branching to nutrient-rich patches, chemical modifications of the rhizosphere, interaction with microorganisms, and engagement of transporters of different parameters, e.g., affinity, are among the most important mechanisms of effective nutrient uptake from a heterogeneous soil environment [27,32,33,34]. One piece of the nutrient acquisition puzzle is the apoplastic barriers in roots. The endodermis, which is essential to selective nutrient uptake [1,17] modifies its differentiation (via the formation of Casparian bands and suberin lamellae) in response to stress factors [12,35], and also nutrient availability [23]. The transition from the primary to the secondary developmental stage switches the function of the endodermis from an absorptive to protective layer [36,37]. Nutrient uptake is adjusted by balancing the symplastic, apoplastic, and transcellular radial transport pathways [25,26]. 

Our results clearly demonstrate that the differentiation of the exodermis also responds to nutrient deficiency, and the reaction type is nutrient-specific. The establishment of the exodermis in the outer cortex of maize roots was significantly accelerated under –N or –P stress but delayed under –K treatments. Roots of –N or –P maize seedlings had Casparian bands and suberin lamellae much closer to the root tip than the control roots did. The opposite was true for –K plants. The response of the endodermis was similar. Its suberization was enhanced in –N or –P roots but delayed in –K roots. These trends generally correspond with previously documented changes in overall radial water flow in wheat, sunflower, and maize; decreased radial water transport under N, P, or S deficiency and increased transport under K deficiency [31,38,39,40]. However, there are also studies showing the opposite trends, e.g., decreased root hydraulic conductivity in low–K barley [41]. Various factors modulate the radial water flow, e.g., the activity of aquaporins and membrane fluidity [38], but reinforcement of the apoplastic barriers with hydrophobic suberin significantly reduces root water permeability [15,16,42]. 

The progress of differentiation depends, in general, on root growth rate. The balance between proliferation, cell elongation, and differentiation, is modulated by environmental stimuli [43,44,45]. Slow growth is commonly connected with the presence of differentiated tissues closer to the root tip [46] and the progress of exodermal differentiation is correlated with root growth rate in some studies as well [8,29]. In our experiments, however, the lengths of –N or –P roots were not significantly affected or tended to be higher in –N conditions (e.g., in –N root compared to +N root in split root hydroponics). –K roots were shorter, but differentiation of endo- and exodermis was postponed into the region further from the root tip. Therefore, the shift in exo- and endodermal differentiation is not simply a matter of growth rate, but the functional response of roots leading to the adjustment of transport properties.

### 3.2. Exodermis and Nutrient Uptake

Exodermal Casparian bands reduce the exposure of plasma membranes to soil solution reaching the middle cortex via the apoplast. Exodermal suberin lamellae may further limit the uptake of compounds entering root apoplast from rhizosphere to rhizodermis only, as the uptake from apoplast to symplast in exodermal cells is prevented by the suberin layer [4,20,21]. Delayed restriction of the apoplastic connection between soil solution and cortex might thus be considered beneficial under nutrient limiting conditions and an increased surface of plasma membrane might extend the interface for nutrient and water uptake. This view was proposed in some studies based on observations of milder outer cortex suberization in less nutritive conditions, e.g., in *Ricinus communis* or *Oryza sativa* roots [20,28]. Enhanced differentiation of exodermis in –N or –P maize roots seems contradictory to this view. We therefore extended our examination to barley with the same result of enhanced exodermal differentiation in -P compared to control conditions. Recently, this response of barley was presented in another study [31]. 

We have analysed the possible involvement of cortical cells in P uptake from the rhizosphere by monitoring the presence of high-affinity Pi transporters (HvPht1;1-2) in cortical and rhizodermal plasma membranes in barley. We could detect HvPht1;1-2 transporters in -P roots only, in accordance with their role in high-affinity Pi acquisition [47]. In a very young, apical part of the root, HvPht1;1-2 occurred in rhizodermal and cortical membranes, including cells of the middle cortex. In older root segments (already at the ¼ position of the length from the tip), the occurrence of transporters was limited to the rhizodermis and exodermis only. This shift even preceded the establishment of lignified exodermal Casparian bands to some extent. 

All these results taken together show that the examined grass crop species respond to severe N or P limitation (zero N or P; strong signs of deficiency on aerial parts) by enhanced differentiation of apoplastic barriers, both in the exodermis and endodermis, in our study. This can be a way to regulate water transport [31,48]. Low hydraulic conductivity of deficient roots might adjust water uptake to higher root/shoot ratio of deficient plants [31]. However, we rather propose that this response might be the preference to prevent nutrient leakage from the stele and cortex back to the rhizosphere over leaving the apoplastic path free for nutrient uptake from the rhizosphere. In strongly nutrient deficient conditions, moving nutrients inward to the cortex via the apoplast would probably not be highly effective. The preferential localization of transporters in the apical part of roots, functioning in yet undepleted soil, may contribute to uptake efficiency, especially for nutrients with low soil mobility [49,50]. From this point of view, the enhanced exodermal differentiation under nutrient limitation is not contradictory, but contributes to preferential allocation of uptake to root apical parts [51], while keeping apoplastic path closed in older root zones. 

The conclusion about changes in root transport characteristics is further supported by the split-root experiments. When split into two chambers with different N availability, maize roots preferentially branched in the N-rich zone, in agreement with previous studies [52]. In addition to supressed branching, the –N roots displayed enhanced differentiation of the exodermis and endodermis compared to +N root of the same plant. This is the first report of a localized response to nutrient deficiency at the level of root apoplastic barriers. The response is executed at the level of individual roots, rather than localized on one side of a single root. Uneven N application to one side of a root (in the “sandwich” cultivation) did not cause any asymmetry in the establishment of apoplastic barriers. However, such asymmetry can be induced by abiotic stress factors, e.g., cadmium toxicity or drought [53]. We also cannot completely exclude some N diffusion from control to –N agar slabs, which might comprise to the negative results of “sandwich” cultures. In spite of that, our results indicate that root systems respond to heterogenous soil nutrient availability, not only by localized branching to nutrient-rich patches [52,54,55,56], but also by the local adjustment of apoplast permeability according to actual nutrient availability in the surrounding rhizosphere. This may help to preferentially allow nutrient uptake in the root apex and balance the benefits of nutrient uptake with the risk of nutrient leakage in older root segments. This opens an interesting general question about the extent of nutrient leakage from living roots and possible variability along the root axis and rhizosphere conditions for root growth. The significance of nutrient leakage was proposed in studies dealing with the nutrient availability and apoplastic barrier differentiation [23,31] and the role of endodermal suberization in the regulation of solute transport in both directions was recently highlighted [48]. Although a substantial efflux of N or P was documented in some studies [57,58,59,60], this phenomenon requires deeper investigation and is a compelling avenue for future research.

We can also imagine that preferential water uptake by roots located in N– or P–sufficient soil patches might stimulate the mass flow of water and nutrients towards surface of these roots and thus enhance the effectivity of overall nutrient acquisition by the plant.

### 3.3. K Deficiency and Variable Response among Species

Interestingly, the low–K response was quite the opposite of what was observed for other nutrient deficiencies and does not fit to the model proposed above. We found that exodermal and endodermal differentiation clearly occurred further back from the root tip in –K maize plants. Similarly, in split-root hydroponics, exodermis with Casparian bands differentiated later in –K roots compared to +K roots of the same plant. Again, we see the response driven by conditions of the surrounding rhizosphere. 

The difference between low–N, low–P, and low–K responses can only be partially explained by the different soil mobility of the dominant plant-accessible forms of these macronutrients. PO_4_^3−^ is not very mobile in soil and a depletion zone often forms in the rhizosphere [49,50]. It is preferentially taken up in the apical part of the root, as indicated by short-term ^33^Pi incubations or expression patterns of high-affinity Pi transporters [61,62]. P acquisition also often relies on associations with mycorrhizal fungi [63], which seems less important for K uptake [64]. NO_3_^−^ is highly mobile and may occur in deeper soil layers due to leaching [49]. Enhanced elongation of main root axis is a typical response to N limited conditions [33,46,62,65]. Preferential targeting of uptake to root apical parts growing in not yet depleted soil or deeper soil layers is thus reasonable for both N and P. K^+^ is rather mobile, mostly present in top soil [49], and low–K induced changes in root system architecture are not uniform, and vary even between ecotypes [66]. Localized root branching into N-rich and P-rich patches is also much more pronounced than into K-rich zones [32]. 

Moreover, potassium is highly mobile within the plant body [50,67] and the endodermis obviously contributes to the regulation of this mobility. Mutants of *Arabidopsis thaliana* affected in endodermal differentiation display surprisingly mild nutrient phenotypes [68,69], but K uptake is the most affected. For example, the *Shengen3/gassho1* mutant, with disrupted CB definition and no compensatory suberin upregulation, displays symptoms of K-limitation and sensitivity to low K stress. [69]. In contrast, the *esb1* dirigent-protein mutant with disrupted bands, but doubled root suberin, had higher K content in shoot [68].

Moreover, it appears the response of apoplastic barriers to –K stress is species-specific, and in need of further research. A recent study in barley found no changes in root suberization in response to K deficiency [41], while *Arabidopsis thaliana* showed enhanced endodermal suberization under K deficiency, but supressed endodermal suberization under Fe and P deficiencies [23,70]. Differences between species may be related to plant specific factors (e.g., root size, the presence or absence of an exodermis or aerenchyma, the extent of mycorrhiza), but also to differences in experimental design (e.g., real nutrient availability for the whole plant, rhizosphere conditions of individual roots). 

## 4. Materials and Methods 

### 4.1. Experimental Cultivations of Maize

*Zea mays* L. cv. Cefran (supplier: Oseva Bzenec, Czech republic) caryopses were germinated on moistened filter paper. Then, 4-DAG (days after germination) seedlings (with app. 3 cm primary root length, no laterals) were transferred into the experimental setup. 

The nutrient-specificity of the response was tested in hydroponic culture (Figure 1a). Seedlings were transferred to 12 L plastic containers (6 plants per container) in a cultivation room (16/8h day/night regime; irradiance 435 W m^−2^, 22/18 °C day/night, relative humidity 50%–75%). Plants grew in aerated quarter-strength Hoagland solution (C, control treatment) and six deficient treatments, each lacking one of following nutrients: N, P, K, Ca, Mg, Fe (for detailed composition see Table 1). The cultivation period was 14 days and the nutrient solution was changed once (after 7 days of cultivation). The aeration was provided by standard aquarium pumps.

The local effects of nutrient deficiencies on root development were tested in split roots hydroponics and agar “sandwich” cultivations, which provided independent cultivation to support results gained from the conventional hydroponics. 4-DAG seedlings were pre-cultivated for 2 days in quarter-strength Hoagland solution (control treatment; Table 1) and all roots, except two equal adventitious seminal roots (ca. 5 cm), were removed. Retained roots were distributed into two 6 L chambers (5 plants per container) of an aerated split-root hydroponic system (Figure 3a) and cultivated for 10 days. Three deficiencies were tested (–N, –K, and –Fe) in the following arrangement: control/control (C/C), control/deficient (C/–N, C/–K, and C/–Fe), and deficient/deficient (–N/–N, –K/–K, and –Fe/–Fe). In control/-N treatment, C/-N(C) marked the root growing in the control chamber and C/–N(–N) marked the root of the same plant growing in –N chamber (the same arrangement applied for C/–Fe and C/–K treatments). The nutrient composition of treatments was as described in Table 1.

The agar “sandwich” cultivation, inspired by [10,71], was arranged as in Figure 4a–c. 4-DAG seedlings (with or without caryopses) were placed between two agar slabs (20 × 20 × 0.5 cm; supplemented with 1% agar) with different N availability: control/N deficient (C/-N) and control/control (C/C). Agar slabs were separated by silicone rubber spacers (1.5 cm width; Figure 4a), supported by glass, and placed in a humid chamber to prevent drying of the agar plates (Figure 4c). Plants were harvested 2 days later. 

### 4.2. Anatomical Analyses and Biometric Characteristics 

Sampled roots were fixed in 4% formaldehyde, gently evacuated and hand sectioned to approx. 100 µm using hand-microtome and *Sambucus* pith as the mechanical support [72]. The sections were done at ^1^/_12_, ^1^/_4_, ^1^/_6_, ^1^/_2_, and ¾ of its total length from the root tip to gain segments of comparable age (Figure 2a). Sections were stained with Sudan Red 7B or with berberine hemisulphate counterstained with Crystal Violet [73]. All these stains were purchased from Sigma-Aldrich, Inc. Sections mounted in 65% glycerol were observed with bright field or fluorescence optics (U-MWU filter cube) of Olympus BX51 microscope (Olympus Corp., Tokyo, Japan) and Apogee U4000 digital camera (Apogee Imaging Systems, Inc., Roseville, CA, USA). Development of the exo- and endodermis was quantified according to the frequency of cells with detectable Casparian bands (CB) or suberin lamellae (SL), as described in [5]. The following developmental categories were used: 0 (missing; 0% of cells with detectable CB or SL), I (low; <30% of cells with CB or SL), II (medium; app. 50% of cells with CB or SL), III (high; >70% of cells with CB or SL), and IV (complete; 100% of cells with CB or SL). 

The length of the main axis was recorded for each root during sampling. The shoot length and its dry weight (dried to a constant weight at 60 °C) were recorded. Root length was measured at the start and end of the cultivation period in short-time sandwich cultures. The image analysis of root branching was done with NIS Elements AR 3.22.05 (Laboratory Imaging) software. 

### 4.3. Immunolocalization of Phosphate Transporters in Barley Roots

Caryopses of barley (*Hordeum vulgare* L. cv. Henriette; supplier: Central Institute for Supervising and Testing in Agriculture, Czech Republic) were germinated for 3 days on moistened filter paper and transferred to 12 L plastic containers (6 plants per container) in a cultivation room. Plants were precultivated in aerated quarter-strength Hoagland solution (control treatment; Table 1) for one week, then transferred either into control or P deficient conditions (Table 1) and cultivated two more weeks. The nutrient solution was renewed once within this period. Sampled roots were fixed in 4% formaldehyde in phosphate buffer (0.1mM, pH 7.1) for 2 h, and sectioned at different positions along the root axis. The sectioning was done with hand-microtome, similarly as in maize (see above). Anti-HvPht1;1-2 primary antibody (Agrisera, cat. No. AS08 321; dilution 1:100) and Goat anti-rabbit IgG DyLightTM 488 secondary antibody (Agrisera, cat. No. AS09633; dilution 1:1000) were applied according to Soukup [73]. Anti-HvPht1;1-2 primary antibody cannot discriminate between HvPht1;1 and HvPht1;-2 transporters due to their similarity, we therefore mention them together throughout this text. The antibodies were diluted in 1x PBS (phosphate buffered saline) with 10 mg BSA (bovine serum albumin) per 1 mL of 1x PBS. The primary antibody was excluded from the procedure in negative controls. Parallel sections from the same root axis position were subjected to histochemical detection of CB and SL as described above.

### 4.4. Statistical Analyses

The statistical analysis was done with NCSS 9.0.15 package (Hintze, J. 2013. NCSS, LLC. Kaysville, UT, USA). One-way ANOVA (Bonferroni Test) was used to evaluate variation between the samples. The correlation between root length and barrier differentiation state was analysed using Correlation Matrix (Pearson correlation coefficient).

## 5. Conclusions

In summary, the data presented in this study highlight the developmental plasticity of apoplastic barriers in response to nutrient availability. This response is nutrient-specific and depends on the local conditions of the surrounding rhizosphere. The establishment of the exodermis in the outer cortex of maize roots is significantly accelerated under –N or –P stress but delayed under –K treatments.

## Figures and Tables

**Table 1:** Nutrient composition of control and deficient treatments (mg L^−1^).

	Treatments						
Compounds	C (Control)	–N	–P	–K	–Ca	–Mg	–Fe
Ca (NO_3_)_2_·4H_2_O	295.5	0	295.5	442.7	0	295.5	295.5
KNO_3_	126.5	0	126.5	0	379	126.5	126.5
KH_2_PO_4_	34	34	0	0	34	34	34
MgSO_4_·7H_2_O	61.4	61.4	61.4	61.4	61.4	0	61.4
CaCl_2_·H_2_O	0	162.1	0	0	0	0	0
K_2_SO_4_	0	108.8	21.8	0	0	43.4	0
NaH_2_PO_4_·2H_2_O	0	0	0	38.7	0	0	0
Fe citrate	5	5	5	5	5	5	0
H_3_BO_3_	2.86	2.86	2.86	2.86	2.86	2.86	2.86
MnCl_2_·4H_2_O	0.715	0.715	0.715	0.715	0.715	0.715	0.715
ZnSO_4_	0.453	0.453	0.453	0.453	0.453	0.453	0.453
(NH_4_)_6_Mo_7_O_24_·4H_2_O	0.056	0.056	0.056	0.056	0.056	0.056	0.056
CuSO_4_	0.019	0.019	0.019	0.019	0.019	0.019	0.019
